# Deciphering the complete chloroplast genome sequence of *Meconopsis torquata* Prain: Insights into genome structure, comparative analysis and phylogenetic relationship

**DOI:** 10.1016/j.heliyon.2024.e36204

**Published:** 2024-08-13

**Authors:** Sheikh Sunzid Ahmed, M. Oliur Rahman

**Affiliations:** Department of Botany, Faculty of Biological Sciences, University of Dhaka, Dhaka, 1000, Bangladesh

**Keywords:** Plastome assembly, Nucleotide diversity, *Meconopsis torquata*, Phylogenetics, Molecular dating, Bioinformatics

## Abstract

In the present study, we have characterized the complete chloroplast (Cp) genome of *Meconopsis torquata* Prain (family Papaveraceae), revealing the plastome size of 153,290 bp, and a GC content of 38.72 %. The cp genome features the typical circular quadripartite structure found in flowering plants, including a pair of inverted repeat regions (25,816 bp), isolated by a small single-copy region (17,740 bp) and a large single-copy (83,918 bp). Genome annotation revealed 132 genes: 87 protein-coding genes, 37 tRNAs and eight rRNAs. This comparative study demonstrated that the genome structure, gene number and GC ratio are consistent with several other cp genomes of *Meconopsis* and *Papaver* genera. A total of 120 SSRs were detected in the plastome, the majority (111) of which were mononucleotide repeats. Among the longer repeats, palindromic sequences were most common, followed by forward, reverse, and complement repeats. The whole genome alignment revealed the conserved nature of the inverted repeat region over single-copy zones. Nucleotide diversity unveiled hypervariable sites (*ycf1*, *rps16*, *accD*, *atpB* and *psbD*) in both the small and large single-copy regions, which could be useful for designing molecular markers for taxonomic identification. Phylogenetic analysis revealed a close alliance of *M. torquata* with other *Meconopsis* species, such as *M. pinnatifolia* and *M. paniculata*, with strong bootstrap support. Molecular dating suggests that *M. torquata* originated during the Tortonian age of the Miocene epoch of the Cenozoic era. These findings provide valuable insights for biological research, especially in understanding the genetic and evolutionary divergence within the Papaveraceae family.

## 1 Introduction

Chloroplasts (Cp) characterized by a dual-layered membrane, autonomous DNA, and thylakoid systems, originated from a symbiotic relationship between a photosynthetic bacterium and a non-photosynthetic host, maintaining their unique genetic material [1,2]. Responsible for photosynthesis, these organelles generate energy for plants and algae while aiding in the synthesis of essential metabolites. The typical angiosperm cp genome consists of four parts: a large single-copy (LSC) region, a small single-copy region (SSC), and two inverted repeats (IRs) [3,4]. Chloroplast DNA is non-recombinant in nature and is inherited uniparentally. Compared to mitochondrial and nuclear genomes, chloroplast genomes demonstrate the highest level of conservation in nucleotide sequence, composition, and structure. Consequently, they are widely employed in molecular systematics research. The quantity of chloroplast genomes cataloged and stored in the NCBI (National Center for Biotechnology Information) database is steadily increasing. The utilization of publicly available SRA (Sequence Read Archive) next-generation sequencing data stored in NCBI represents a transformative approach for chloroplast genome assembly, relying on existing datasets rather than generating new wet-lab data. This strategy significantly reduces the time, cost, and resources associated with traditional sequencing efforts. By accessing this vast repository of genetic information, researchers can efficiently reconstruct complete chloroplast genomes with remarkable accuracy and depth [5,6]. Leveraging public data not only enhances the scalability and accessibility of chloroplast genome assembly but also promotes collaboration and reproducibility across scientific communities. This democratization of genomic data has fostered innovation and accelerated discoveries in plant biology and beyond. Therefore, the use of public data for chloroplast genome assembly represents a paradigm shift in genomics research, unlocking new avenues for exploration and advancing our current understanding of plant evolution, ecology, and adaptation [7].

*Meconopsis* Viguier, a genus of the Papaveraceae family known for its medicinal and economic significance, occurs in alpine environments and thrives on the shaded slopes of rocky mountain ranges [8]. Typically, *Meconopsis* can be detected at elevations between 3600 and 5200 m, although certain variations may also exist at lower altitudes, with a distinct preference for montane habitats. *Meconopsis* encompasses 54 species worldwide, with 53 predominantly located in the Sino-Himalayan region. *Meconopsis torquata* has long been utilized in folk medicine for treating liver heating, lung heating, and laryngitis [9,10]. This species boasts a rich reservoir of bioactive phytoconstituents, presenting promising avenues for drug design and modern pharmaceutical discovery [11]. The medicinal properties of this species are rooted in centuries of traditional knowledge, underscoring its potential significance in contemporary healthcare practices. Recent reports indicate that in China, this species faces various threats, including overexploitation, habitat alteration, and human disturbance [10]. The precarious situation of this species underscores the urgent need for its conservation. Conserving *M. torquata* is crucial not only for safeguarding its genetic diversity and ecological significance but also for maintaining the delicate balance of the ecosystems it inhabits. Moreover, the assembly of the chloroplast genome of *M. torquata* holds immense value for understanding its evolutionary history, population dynamics, and genetic adaptation to environmental challenges. By elucidating the cp genome architecture of *M. torquata*, it will be possible to devise more effective identification strategies and conservation measures tailored to its specific genetic makeup and ecological requirements, thereby ensuring its survival for future generations [12,13]. Additionally, leveraging the unique features of the cp genome, such as its high copy number and stable expression platform, offers opportunities for utilizing *M. torquata* in biotechnological applications [[14], [15], [16], [17]].

There has been no detailed study on the complete plastome of *M. torquata* so far, resulting in a lack of essential information needed to accurately determine its phylogenetic relationships and divergence time using appropriate barcodes. Therefore, the objectives of this investigation are to present the complete cp genome of *M. torquata* and analyze its genomic features using comparative phylogenetic and molecular dating approaches. This study also aims to identify the promising DNA barcodes for the molecular identification of *M. torquata* and enhance our current understanding of its systematic position within Papaveraceae family. Molecular dating efforts can illuminate the timing of divergence in accordance with the geological timescale. This endeavor will significantly contribute to the genetic classification and evolutionary study of *M. torquata*.

## 2 Materials and methods

### 2.1 Retrieval of NGS data and quality assessment

The SRA repository of the NCBI was accessed to retrieve short-read sequencing data of the *M. torquata* chloroplast genome using the SRA accession “SRR27499558”. Next-generation sequencing (NGS) data were extracted with the fastq-dump tool of the SRA-toolkit and subsequently subjected to quality assessment via the FASTQC tool v.0.12.1. After validation, the cleaned paired-end reads were forwarded for plastome assembly and annotation.

### 2.2 Assembly and annotation of the plastome and comparison with existing sequence

The raw reads of *M. torquata* were assembled using the GetOrganelle v.1.7.7.0 pipeline to generate the plastome [18]. *M. racemosa* (GenBank accession OL790391.1) was used as the seed sequence and GetOrganelleDB 0.0.1 was used as the reference database. The other parameters used were the same as those used in the GetOrganelle pipeline. Afterwards, the assembled fasta genome was mapped against the raw reads using the BWA-MEM module in the Unipro UGENE software for coverage analysis [19]. The circularized plastome was annotated using the GeSeq server with the default parameter settings. The output generated by GeSeq was checked manually to correct gene coordinates, internal stop codons, and open reading frames [20]. A circular map of the plastome was constructed using the volcano coloring scheme in the Chloroplot server [21]. After assembly and annotation, the plastome was submitted to NCBI with the accession number TPA: BK065168.

The assembled sequence of the present study (BK065168) was aligned with the existing GenBank sequence (PP112995.1) of *M. torquata* using MEGA v.11 [22] for comparative analysis. The alignment was carefully examined for SNP (Single Nucleotide Polymorphism) assessment. The DnaSP v.5 software was used to analyze insertion-deletion events between the two plastomes [23]. Synteny analysis was subsequently conducted using the Circoletto server to evaluate concordance between the two Cp genomes [24].

### 2.3 Repeat sequences and codon usage assessment

The presence of repeat elements within the chloroplast genome of *M. torquata* was examined utilizing two distinct servers. The MIcroSAtellite identification tool [25] was employed to detect Simple Sequence Repeats (SSRs). Additionally, the REPuter program [26] was used to identify longer repeat sequences. Relative synonymous codon usage (RSCU) calculations were conducted using MEGA v.11 software [22].

### 2.4 Inverted repeat (IR) expansion, genome divergence and nucleotide diversity

Quadripartite junction sites and the genes at junction sites were analyzed to understand the expansion of inverted repeats employing the IRscope server [27]. The manually curated GenBank file of *M. torquata* was uploaded to the server, whereas GenBank accession numbers were used for other taxa. The Cp genomes used for IR analysis included *M. torquata* (BK065168), *M. racemosa* (OL790391.1), *M. punicea* (NC_050878.1), *P. somniferum* (OM174296.1), *P. orientale* (NC_037832.1), and *P. rhoeas* (NC_037831.1). The plot on the server was generated to assess the expansion and contraction of the inverted repeat region.

For genome divergence analysis, the aforementioned taxa were used along with their corresponding GenBank accession numbers, with. *M. torquata* (BK065168) as the reference. The sequences were retrieved from NCBI and aligned using the mVISTA server. The analysis in mVISTA [28] was conducted using the Shuffle-LAGAN mode.

Nucleotide diversity analysis was performed by aligning the cp genome sequences initially in the MAFFT server [29]. DnaSP v.5 software was utilized for estimation of the nucleotide variation [23]. The parameters for window length and step size were defined as 600 base pairs and 200 base pairs, respectively. A comparative analysis of genomic coordinates for each window was conducted against gene annotations of the chloroplast genome to elucidate the characteristics of nucleotide diversity indices. To determine the most potential DNA barcode following nucleotide diversity, the hypervariable genes were analyzed. A Neighbor-joining (NJ) tree with 1000 bootstrap replicates was constructed using the Poisson model in MEGA v.11. Gamma distribution rates were chosen, and partial deletions were selected for missing data treatment [22].

### 2.5 Molecular phylogenetic analysis

*Sabia swinhoei* Hemsl. (NC_069951.1) and *Pachysandra terminalis* Siebold & Zucc. (MZ636535.1) were chosen as outgroup taxa for the molecular phylogenetic analysis. Initially, chloroplast genomes of 49 taxa were retrieved from the GenBank database to establish a plastome-wide molecular phylogeny. These sequences were aligned using the MAFFT server and the resulting output file, formatted in fasta, was utilized for Maximum-Parsimony (MP) analysis in MEGA v.11 software. The MP tree was generated employing the Subtree-Pruning-Regrafting approach.

### 2.6 Molecular dating endeavor

The molecular dating analysis was conducted using the Clocks module within MEGA v.11 [22]. Specifically, the RelTime-ML submodule within the Clocks module was utilized for this study. Initially, the MAFFT alignment file of the Cp genomes was loaded into the software. Subsequently, the outgroup taxa were defined, and calibration nodes were established by consulting the TimeTree server, selecting five nodes based on the available taxa [30].

## 3 Results

### 3.1 Assembly and annotation of the cp genome and comparison with existing sequence

The FASTQC tool revealed a GC content of 38 % for the forward-end reads and 39 % for the reverse-end reads, with a sequence length of 150 bp. The raw reads exhibited satisfactory results for all the quality-evaluating parameters including per base sequence quality, per tile sequence quality, per sequence quality scores, and sequence length distribution (Supplementary Figs. S1 and S2). The per base sequence quality analysis for both forward and reverse reads indicated high-quality sequencing data. The forward reads consistently had mean quality scores exceeded 35, with a median score of 37 across all bases and a tight distribution around the median, demonstrating exceptional sequencing accuracy and consistency (Supplementary Table S1). Similarly, the reverse reads exhibited mean quality scores ranging from 33.77 to 36.19, with a median score consistently at 37 and minimal variability, despite a slight decline towards the end (Supplementary Table S2). Both reads showed uniformly high-quality scores with 10th and 90th percentiles at 37 for most positions, confirming robust data quality. The initial variability in the reverse reads quickly stabilized, and both read sets passed the FASTQC quality checks, ensuring that the sequencing data was highly reliable and suitable for downstream analyses. Therefore, no trimming endeavors were deemed necessary for the raw reads.

**Fig. 1:**
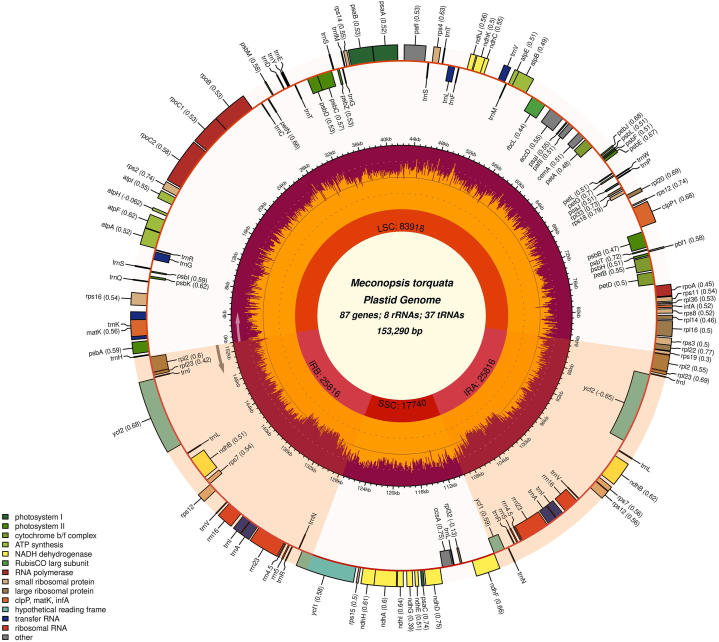
Genome map of the complete chloroplast genome of *M. torquata*. The shaded areas represent regions of inverted repeats. Codon usage bias for each protein-coding gene is indicated in parentheses next to the gene name.

The plastome was successfully constructed into its circularized form via *de novo* assembly. The 153,290 bp plastome exhibited the characteristic quadripartite structure with a large single-copy (LSC) region (83,918 bp), a small single-copy (SSC) region (17,740 bp) and two inverted repeat regions (25,816 bp each) (Fig. 1). After mapping the cp genome, coverage analysis revealed a mean coverage depth of 2445.15×, with a minimum depth of 791× and a maximum depth of 3452×. The GC content in the assembled cp genome was 38.72 %, and the base frequencies were 30.37 % (A), 30.91 % (T), 19.77 % (C) and 18.95 % (G). The GC content in the SSC, LSC, and IR regions was 33.12 %, 37.23 %, and 43.1 %, correspondingly. The SSC and LSC regions had a higher percentage of A and T (U) bases compared to the IR regions. Conversely, the IR regions exhibited a higher percentage of C and G bases compared to the SSC and LSC regions (Table 1).

**Table 1:** Composition of nucleotides in the cp genome of *Meconopsis torquata*.

Region	A (%)	T (U) (%)	C (%)	G (%)	C + G (%)	A + T (%)
cp genome	30.37	30.91	19.77	18.95	38.72	61.28
LSC	30.85	31.92	19.12	18.11	37.23	62.77
SSC	33.59	33.30	17.71	15.41	33.12	66.89
IRA	28.20	28.70	22.16	20.94	43.1	56.9
IRB	28.70	28.20	20.94	22.16	43.1	56.9

The plastome contained a comprehensive set of 132 genes, comprising 37 tRNAs (transfer RNA), 8 rRNAs (ribosomal RNA), and 87 protein-coding genes (PCGs), as depicted in Fig. 1. Among the PCGs, 44 were identified as photosynthesis-related, with 19 specifically linked to photosystem I and II functions. The functional emphasis on transcription and translation highlighted 76 genes, primarily comprising tRNAs. Among these genes, 26 were associated with ribosomal components, encompassing both small (15) and large (11) subunits. Gene duplication was notably prominent within rRNAs, with equal duplications observed across all the rRNAs (Table 2). In the inverted repeats region, tRNAs and rRNAs prevailed, while the SSC region exhibited a prevalence of NADH dehydrogenases. Two notable DNA barcodes, such as *rbcL* and *matK* were identified within the LSC region. Additionally, the LSC harbored the gene *cemA*, encoding the cp envelope membrane protein. Photosystem I and II assembly factors (*pafI* and *pafII*) shared their positions in the LSC, where *pafI* displayed a counter-clockwise direction and *pafII* showed a clockwise-direction for translation.

**Table 2:** Functional classification and gene content of *Meconopsis torquata* cp genome. Multiple copies of genes are marked in parenthesis.

Name of genes	Group of genes	Category
*rrn4.5* ( × 2)*, rrn5* ( × 2)*, rrn16* ( × 2)*, rrn23* ( × 2)	Ribosomal RNA genes (rRNA)	RNA genes
*trnA-UGC* ( × 2)*, trnC-GCA, trnD-GUC, trnE-UUC, trnF-GAA, trnG-GCC, trnG-UCC, trnH-GUG, trnI-CAU* ( × 2), *trnI-GAU* ( × 2), *trnK-UUU, trnL-CAA* ( × 2)*, trnL-UAA, trnL-UAG, trnM-CAU, trnN-GUU* ( × 2)*, trnP-UGG, trnQ-UUG, trnR-ACG* ( × 2)*, trnR-UCU, trnS-UGA, trnS-GGA, trnS-GCU, trnT-UGU, trnT-GGU, trnV-UAC, trnV-GAC* ( × 2)*, trnW-CCA, trnY-GUA, trnfM-CAU*	Transfer RNA genes (tRNA)	
*rps2, rps3, rps4, rps7* ( × 2)*, rps8, rps11, rps12* ( × 3)*, rps14, rps*15, *rps16, rps18, rps19*	Small subunit of ribosome	Ribosomal proteins
*rpl2* ( × 2)*, rpl14, rpl16, rpl20, rpl22, rpl23* ( × 2)*, rpl*32, *rpl33, rpl36*	Large subunit of ribosome	Transcription genes
*rpoA, rpoB, rpoC1, rpoC2*	DNA dependent RNA polymerase	
*psaA, psaB, psaC, psaI, psaJ*	Photosystem I	Protein genes
*psbA, psbB, psbC, psbD, psbE, psbF, psbH, psbI, psbJ, psbK, psbL, psbM, psbT, psbZ*	Photosystem II	
*petA, petB, petD, petG, petL, petN*	Subunit of cytochrome	
*atpA, atpB, atpE, atpF, atpH, atpI*	Subunit of synthase	
*rbcL*	Large subunit of rubisco	
*ndhA, ndhB* ( × 2)*, ndhC, ndhD, ndhE, ndhF, ndhG, ndhH, ndhI, ndhJ, ndhK*	NADH dehydrogenase	
*clpP1*	ATP-dependent protease subunit P	
*cemA*	Cp envelope membrane protein	
*matK*	Maturase	Other genes
*accD*	Subunit acetyl-coA carboxylase	
*ccsA*	C-type cytochrome synthesis	
*Pbf1*	Photosystem biogenesis factor 1	
*ycf1* ( × 2)	Component of TIC complex	
*ycf2* ( × 2)	Hypothetical proteins	
*pafI, pafII*	Photosystem I and II assembly	
*infA*	Translation initiation factor	

**Fig. 2:**
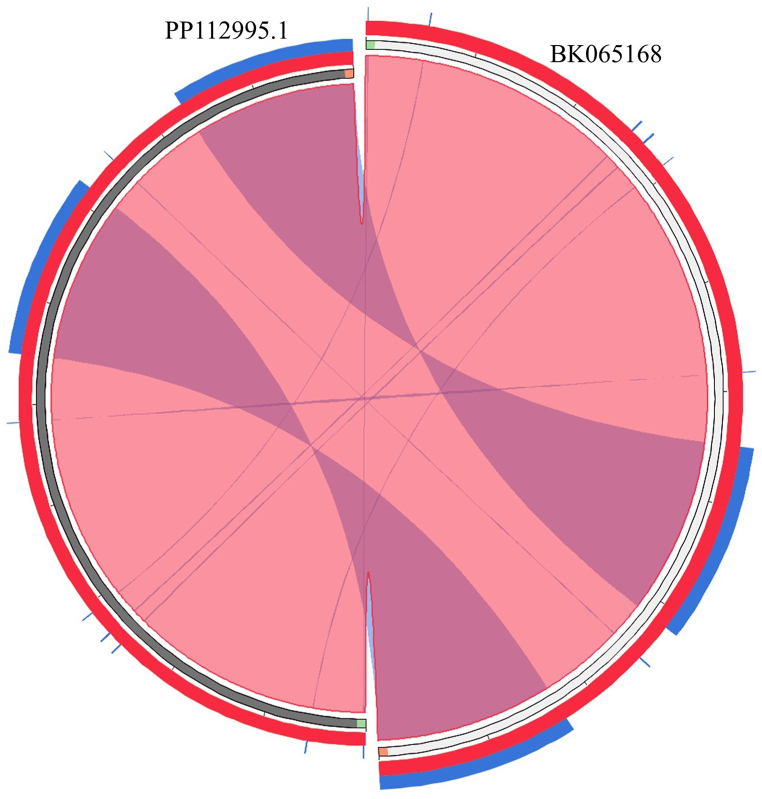
Comparative analysis using Synteny approach, demonstrating the high similarity between the assembled sequence (BK065168) and the existing cp genome (PP112995.1).

The comparative assessment revealed a high similarity between the assembled plastome (BK065168) and the existing sequence of *M. torquata* (PP112995.1). The whole genome alignment displayed 100 % similarity, with no single nucleotide polymorphisms detected. Insertion-deletion analysis showed no insertion-deletion events between the two plastomes. Synteny analysis revealed a lack of large rearrangements and a high similarity between the two plastomes (Fig. 2). These comparative evidences reinforce the reliability and robustness of our assembly process and confirm the accuracy of current results.

### 3.2 Repeat structure and codon usage analysis

**Fig. 3:**
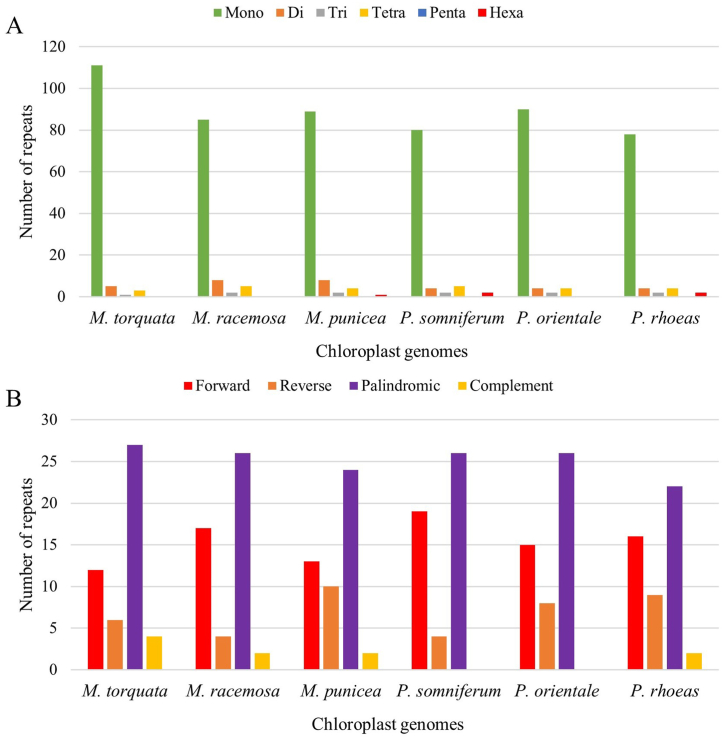
Comparative analysis of repeat structures across various cp genomes. A. Simple sequence repeats, B. Longer repeats.

In the plastome of *M. torquata*, 120 SSRs were identified, with the highest frequency observed in mononucleotide repeats (111), followed by dinucleotide, tetranucleotide and trinucleotide repeats (Fig. 3A). No penta- or hexanulceotide repeats were detected in the cp genome of *M. torquata*. Comparative analysis of SSR repeats revealed a very similar pattern in other closely related taxa. The plastome of *M. racemosa* lacked both penta- and hexanucleotide repeats, while the cp genome of *M. punicea* lacked only pentanulcleotide repeats. All *Papaver* species exhibited similar results. Additionally, 49 longer repeat structures were detected in the *M. torquata* plastome and were categorized into four major types: forward (12), reverse (6), palindromic (27) and complement (4) repeats (Fig. 3B and Supplementary Table S3). The distribution of longer repeats across various cp genomes was similar, with the highest frequency observed for palindromic repeats, followed by forward, reverse, and complement repeats. This similarity in repeat analysis further validated the assembly of *M. torquata* cp genome.

Codon usage analysis unveiled the utilization of 64 distinct codons encoding 20 unique amino acids, with a total codon frequency of 51,096 (Supplementary Table S4). Among these, arginine (CGC) exhibited the lowest codon frequency (255), closely trailed by alanine (GCG). In contrast, phenylalanine (UUU) boasted the highest frequencies (2,051) followed by lysine (AAA) (1987). The RSCU (relative synonymous codon usage) values of individual codons ranged from 0.45 to 1.93, indicating varying degrees of usage bias. Since different codons can encode the same amino acid, the RSCU values varied accordingly for amino acids. The cumulative RSCU value peaked at 6.01 for arginine and serine, with leucine closely trailing at 6.00. Interestingly, 31 codons showed usage frequencies exceeding the expected equilibrium (RSCU >1), while an equal number showed usage bias (RSCU <1). Notably, AUG (methionine) and UGG (tryptophan) demonstrated unbiased usage, both with RSCU values of 1.

The codon usage analysis across five species, including *M. racemosa*, *M. punicea*, *P. somniferum*, *P. orientale*, and *P. rhoeas*, revealed distinct patterns in RSCU values and total codon frequencies. *M. racemosa* exhibited a total codon frequency of 51,337, with individual codon RSCU values ranging from 0.42 to 1.82. Notably, leucine, arginine, and serine showed high RSCU values of 5.99, 6.01, and 6.0, respectively, indicating preferred codon usage. Methionine (AUG), tryptophan (UGG), and glycine (GGG) displayed unbiased codon usage (RSCU = 1) in this species. *M. punicea* showed a slightly lower total codon frequency of 51,093, with RSCU values ranged from 0.46 to 1.92. The RSCU values for leucine, arginine, and serine were found to be 5.99, 6.0, and 5.99, respectively. Unbiased codon usage was observed for methionine (AUG), tryptophan (UGG), and the stop codon (UGA). *P. somniferum* showed a total codon frequency of 50,985, with individual RSCU values spanning from 0.44 to 1.88. The RSCU values for leucine, arginine, and serine were each 6.0, 6.0, and 6.01, respectively. This species exhibited unbiased codon usage for methionine (AUG) and tryptophan (UGG). For *P. orientale*, the total codon frequency was 50,933, with RSCU values ranging from 0.47 to 1.83. The RSCU values for leucine, arginine, and serine were consistently 6.0. The unbiased codon usage was observed for methionine (AUG) and tryptophan (UGG) in this species. Lastly, *P. rhoeas* had a total codon frequency of 50,968, with RSCU values varied from 0.48 to 1.81. The RSCU values for leucine, arginine, and serine were 6.0, mirroring the pattern seen in *P. orientale*. Unbiased usage for methionine (AUG) and tryptophan (UGG) was similarly observed in *P. rhoeas*. Across all species, leucine, arginine, and serine exhibited high cumulative RSCU values, indicating a significant codon usage bias for these amino acids.

### 3.3 Inverted repeat (IR) expansion and contraction analysis

**Fig. 4:**
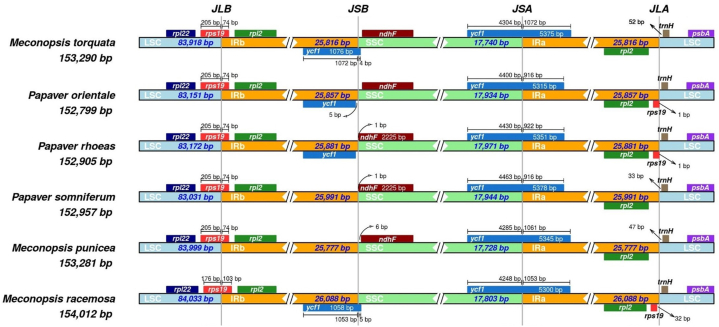
Quadripartite structure and junction sites among the LSC, IR and SSC regions of *M. torquata* and other cp genomes. The numeric values positioned above or adjacent to the colored genes denote the distances between each gene and the border edges.

The junction site analysis revealed a high degree of similarity in size, organization, and gene features among the studied taxa (Fig. 4). The sizes of the LSC, SSC, and IR regions of all five species were very similar to those of the *M. torquata* cp genome. The LSC varied from 83,031 to 84,033 bp, and the SSC ranged from 17,728 to 17,971 bp. IRa and IRb also displayed variation within a narrow range. In all the taxa examined, *rps19* was found at the junction of LSC/IRb when the translation direction was from IRb to LSC. In *M. torquata*, *rps19* gene originated from IRb and extended to the LSC region, with a 205 bp overlap in the LSC region. This pattern was consistently observed in the cp genomes of *M. punicea*, *P. somniferum*, *P. rhoeas* and *P. orientale*. When translated from IRa to LSC, the *rps19* gene formed a 1 bp overhang in the LSC/IRa junction for both *P. orientale* and *P. rhoeas*. The *rpl22* gene was located entirely within the LSC region across all the studied species. Likewise, *ndhF* was exclusively found within the SSC region across all taxa except for *M. racemosa.*

The *ycf1* gene was found to be closely parallel across all taxa when translated from the complementary strand, showing a distinct demarcation between the IRa and SSC regions. Originating from IRa in the complementary strand, this gene expanded towards the SSC region, forming an overlapping zone ranging from 4248 to 4463 bp. However, when translated from IRb to SSC, *ycf1* formed a very narrow overhang area. In *M. torquata*, this narrow zone displayed a 4 bp overhang, and in *M. racemosa,* it had an 5 bp overhang. Notably, when considering translation from IRb to SSC, *ycf1* did not expand from IRb to SSC in two taxa, *i.e.*, *P. orientale* and *P. rhoeas,* and it was absent in *P. somniferum* and *M. punicea.* The *rpl2* gene was entirely present in both IRb and IRa, considering IRb to LSC and IRa to LSC directions modes. Finally, *trnH* was entirely located in the LSC region across all the studied taxa.

### 3.4 Genome divergence and comparative assessments

**Fig. 5:**
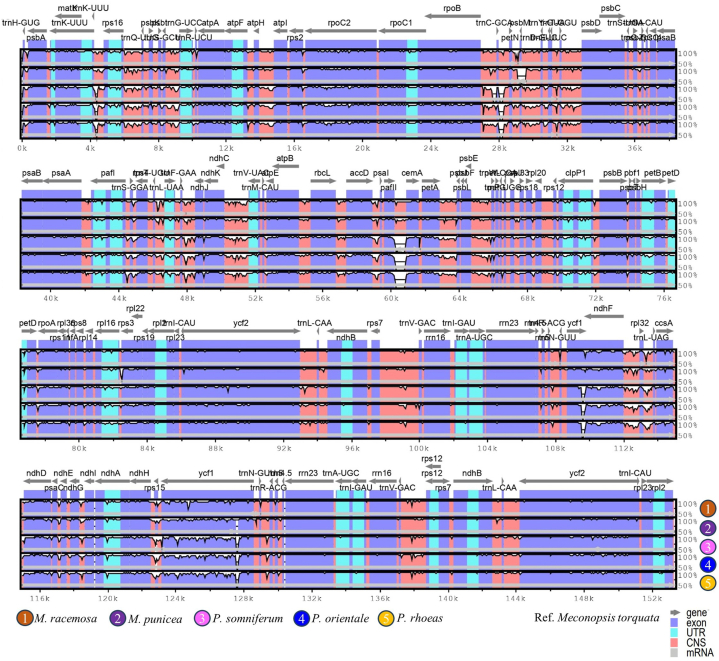
mVISTA genome divergence and percent identity plot representing the comparative positions and gene order of *Meconopsis racemosa*, *M. punicea*, *Papaver somniferum*, *P. orientale* and *P. rhoeas* with *M. torquata* as the reference genome.

The plastome of *M. torquata* served as a reference for elucidating genome divergence among closely related species of *M. torquata*. mVISTA analysis revealed strikingly similar gene orientations and genome organizations across all the examined cp genomes (Fig. 5). Notably, genome divergence was lower within IRa and IRb than in their LSC and SSC counterparts. Within the coding regions, sequences exhibited higher levels of conservation, whereas non-coding regions showed greater variation. As part of the comparative assessment, Table 3 highlights the genomic characteristics of several other closely related taxa used in this study.

**Table 3:** Chloroplast genome characteristics of the taxa investigated in the present study.

Taxa	Accession Number	Family/Tribe	Cp genome (bp)	GC content (%)	PCGs	tRNAs	rRNAs	Total Genes
*Chelidonium majus*	NC_046829.1	Chelidonieae	159,734	38.7	85	36	8	129
*Corydalis filistipes*	MK264349.1	Fumarieae	169,237	41.1	83	37	8	128
*Corydalis impatiens*	NC_060862.1	Fumarieae	197,317	40.6	88	38	8	134
*Corydalis maculata*	MK264348.1	Fumarieae	165,066	40.9	81	37	8	126
*Corydalis namdoensis*	MK264350.1	Fumarieae	169,818	41.0	83	37	8	128
*Corydalis pauciovulata*	MK264352.1	Fumarieae	161,773	41.5	80	35	8	123
*Dactylicapnos grandifoliolata*	NC_088070.1	Fumarieae	176,370	40.1	92	39	8	139
*Dactylicapnos lichiangensis*	NC_088068.1	Fumarieae	175,134	40.5	88	37	8	133
*Dactylicapnos macrocapnos*	NC_088072.1	Fumarieae	175,552	39.9	94	38	8	140
*Dactylicapnos roylei*	OR568572.1	Fumarieae	173,874	40.5	88	37	8	133
*Dactylicapnos scandens*	OR568573.1	Fumarieae	175,605	40.0	94	38	8	140
*Dactylicapnos schneideri*	NC_088071.1	Fumarieae	172,344	40.2	92	39	8	139
*Dactylicapnos torulosa*	NC_088069.1	Fumarieae	174,101	40.6	88	37	8	133
*Dicranostigma lactucoides*	NC_081064.1	Chelidonieae	166,819	39.2	90	36	8	134
*Eomecon chionantha*	NC_079847.1	Chelidonieae	159,849	38.5	88	37	8	133
*Eschscholzia californica*	MK281585.1	Eschscholzieae	160,201	38.7	88	37	8	133
*Fumaria officinalis*	NC_072182.1	Fumarieae	190,247	40.2	96	40	8	144
*Hylomecon japonica*	NC_045388.1	Chelidonieae	160,011	38.8	88	37	8	133
*Hypecoum erectum*	NC_083221.1	Hypecoeae	169,241	38.2	102	49	8	159
*Hypecoum leptocarpum*	NC_081065.1	Hypecoeae	163,282	38.5	77	36	8	121
*Hypecoum zhukanum*	NC_071785.1	Hypecoeae	163,729	38.5	85	39	8	132
*Lamprocapnos spectabilis*	NC_039756.1	Fumarieae	188,754	39.2	98	44	8	150
*Macleaya cordata*	MK281586.1	Chelidonieae	163,107	38.5	89	37	8	134
*Macleaya microcarpa*	NC_039623.1	Chelidonieae	161,118	38.6	86	37	8	131
*Meconopsis bella*	NC_080898.1	Papavereae	153,073	38.9	88	37	8	133
*Meconopsis betonicifolia*	PP542026.1	Papavereae	152,973	38.7	89	37	8	134
*Meconopsis horridula*	PP542027.1	Papavereae	153,914	38.7	89	37	8	134
*Meconopsis integrifolia*	PP542028.1	Papavereae	153,005	38.8	89	37	8	134
*Meconopsis paniculata*	NC_085733.1	Papavereae	152,887	38.7	88	37	8	133
*Meconopsis pinnatifolia*	NC_085732.1	Papavereae	153,557	38.8	86	37	8	131
*Meconopsis pseudohorridula*	NC_061608.1	Papavereae	154,190	38.5	91	37	8	136
*Meconopsis punicea*	NC_050878.1	Papavereae	153,281	38.5	85	37	8	130
*Meconopsis quintuplinervia*	MK801686.1	Papavereae	154,997	38.5	88	37	8	133
*Meconopsis racemosa*	OL790391.1	Papavereae	154,012	38.8	91	37	8	136
*Meconopsis simplicifolia*	NC_070211.1	Papavereae	152,772	38.7	87	37	8	132
*Meconopsis torquata*	BK065168	Papavereae	153,290	38.7	87	37	8	132
*Pachysandra terminalis*	MZ636535.1	Buxaceae	160,772	38.3	88	37	8	133
*Papaver alboroseum*	NC_065204.1	Papavereae	153,703	38.8	88	33	8	129
*Papaver dubium*	NC_065205.1	Papavereae	152,909	38.9	87	32	8	127
*Papaver keelei*	NC_065207.1	Papavereae	153,848	38.8	88	34	8	130
*Papaver mcconnellii*	NC_065209.1	Papavereae	153,687	38.8	88	33	8	129
*Papaver nudicaule*	MW411801.1	Papavereae	152,867	38.8	88	37	8	133
*Papaver orientale*	NC_037832.1	Papavereae	152,799	38.6	88	37	8	133
*Papaver pseudo-orientale*	OM174289.1	Papavereae	152,954	38.6	87	33	8	128
*Papaver rhoeas*	NC_037831.1	Papavereae	152,905	38.8	88	37	8	133
*Papaver somniferum*	OM174296.1	Papavereae	152,957	38.9	87	33	8	128
*Sabia swinhoei*	NC_069951.1	Sabiaceae	161,592	38.6	88	37	8	133
*Sanguinaria canadensis*	NC_079848.1	Chelidonieae	160,910	38.4	88	37	8	133
*Stylophorum lasiocarpum*	MW232434.1	Papavereae	153,196	38.9	88	37	8	133

### 3.5 Nucleotide diversity

**Fig. 6:**
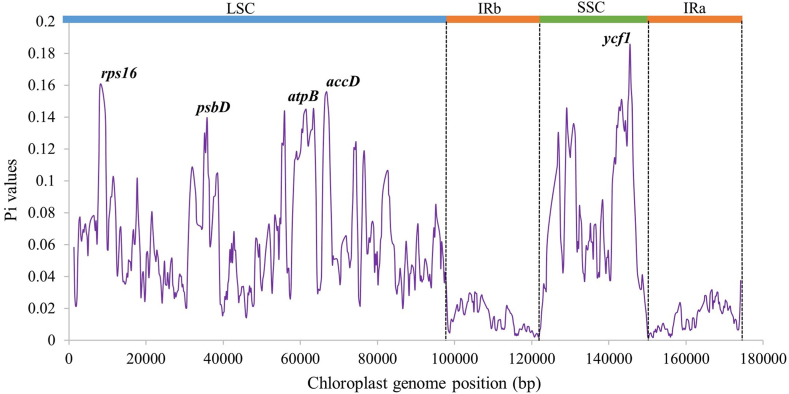
Nucleotide variation across the plastome of *M. torquata* and its close relatives. The length and step of the sliding window are 600 bp and 200 bp, respectively.

Nucleotide diversity within the cp genomes of 10 Papaveroideae taxa revealed distinct patterns of variation. Excluding two outgroup taxa, the average nucleotide diversity (π) was 0.0475, ranging from 0.0012 (*rrn23*) to 0.1857 (*ycf1*). Notably, the SSC and LSC demonstrated significantly greater variability than did the highly conserved inverted repeat (IR) region (Fig. 6), indicating stronger functional constraints on IRa and IRb, thereby limiting sequence divergence. Remarkably, several genes, including *ycf1*, *rps16*, *accD*, *atpB*, and *psbD* displayed exceptionally high π values (>0.1), These hypervariable regions hold promise as molecular marker candidates for taxonomic identification and evolutionary analysis.

**Fig. 7:**
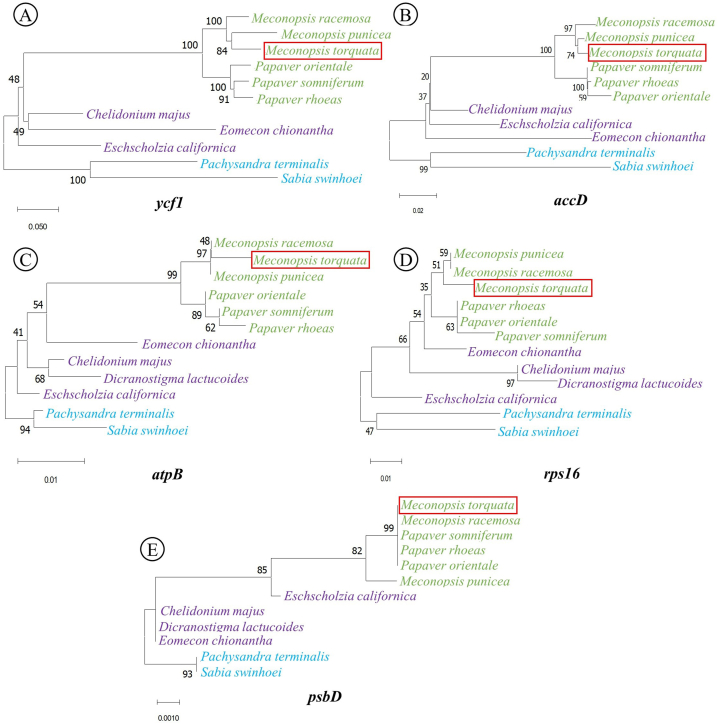
Neighbor-joining tree with 1000 bootstrap replicates showing efficacy of the hypervariable genes within Papaveroideae: A. *ycf1*, B. *accD*, C. *atpB,* D. *rps16*, E. *psbD*.

The hypervariable genes identified from the nucleotide diversity study demonstrated variability in species discrimination efficiency. NJ trees constructed for five genes, viz. *ycf1*, *accD*, *atpB*, *rps16* and *psbD* were depicted in Fig. 7. Among these potential barcodes, *ycf1* exhibited the highest species discrimination efficiency with proper clustering, successfully differentiating all three tribes of the subfamily Papaveroideae. Bootstrap support was higher for the tribe Papavereae compared to other two tribes, Chelidonieae and Eschscholzieae (Fig. 7A). The *accD*, *atpB,* and *rps16* barcodes could differentiate the member taxa of Papavereae but failed to represent the members of Chelidonieae and Eschscholzieae with proper cladding (Fig. 7B–D). The *psbD* gene exhibited the lowest species discrimination efficiency, failing to demonstrate proper clustering in all three tribes (Fig. 7E).

### 3.6 Phylogenetic analysis

**Fig. 8:**
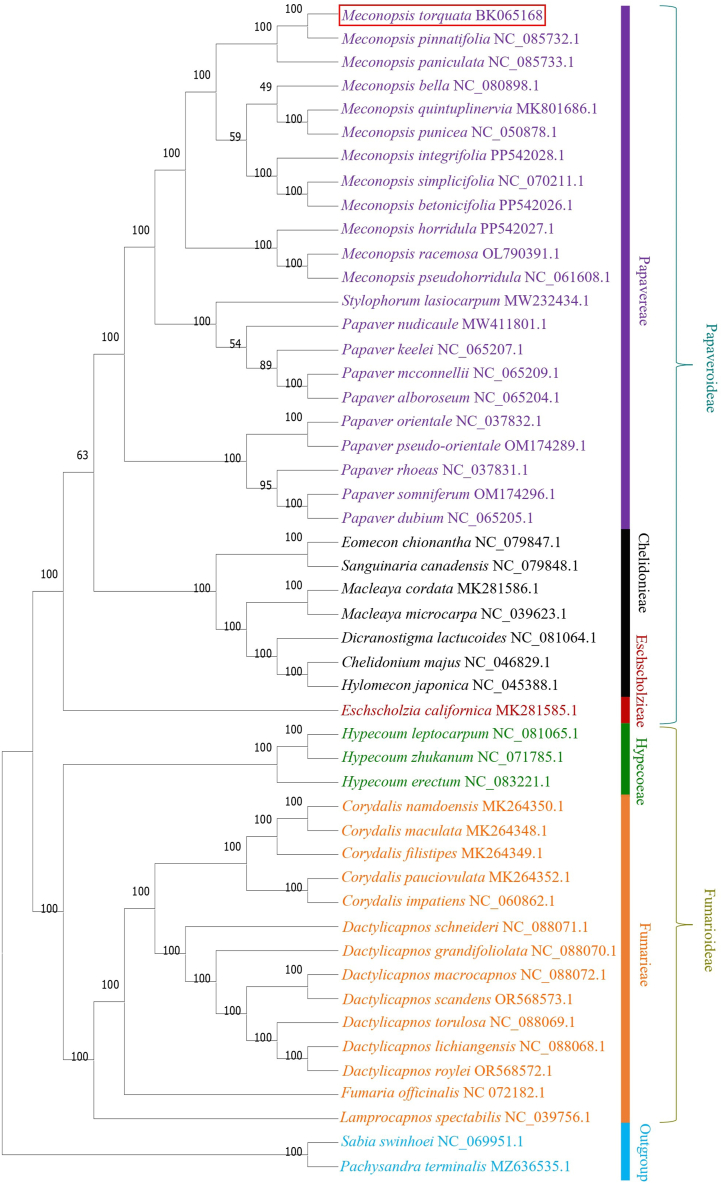
Maximum-parsimony tree with 1000 bootstrap replicates showing phylogenetic relationships of *M. torquata* cp genome within the family Papaveraceae.

The Maximum-Parsimony (MP) approach strongly supported the phylogeny of the member taxa with robust bootstrap values (Fig. 8). The maximum parsimonious tree identified a total of 94,196 evolutionary changes in its total length. The consistency index was 0.749490 for all sites and 0.706695 for parsimony-informative sites. The retention index was 0.923059 for both all sites and parsimony-informative sites. The composite index was recorded as 0.691824 for all sites and 0.652321 for parsimony-informative sites. The two subfamilies of Papaveraceae, Papaveroideae and Fumarioideae, showed clear segregation and distinct clustering. All three tribes of Papaveroideae demonstrated a monophyletic nature. Within the tribe Papavereae, *M. torquata* showed a close relationship with *M. pinnatifolia.* All members of *Meconopsis* demonstrated monophyletic clustering with strong bootstrap support at most nodes.

The genus *Papaver* showed polyphyletic cladding among its member taxa. Within the subfamily Fumarioideae, both Hypecoeae and Fumarieae tribes displayed a monophyletic origin. Similarly, the genera *Corydalis* and *Dactylicapnos* showed monophyly among some other taxa. Bootstrap support was notably stronger in the subfamily Fumarioideae compared to Papaveroideae subfamily, with all nodes in Fumarioideae presenting 100 % bootstrap support.

### 3.7 Molecular dating endeavor

**Fig. 9:**
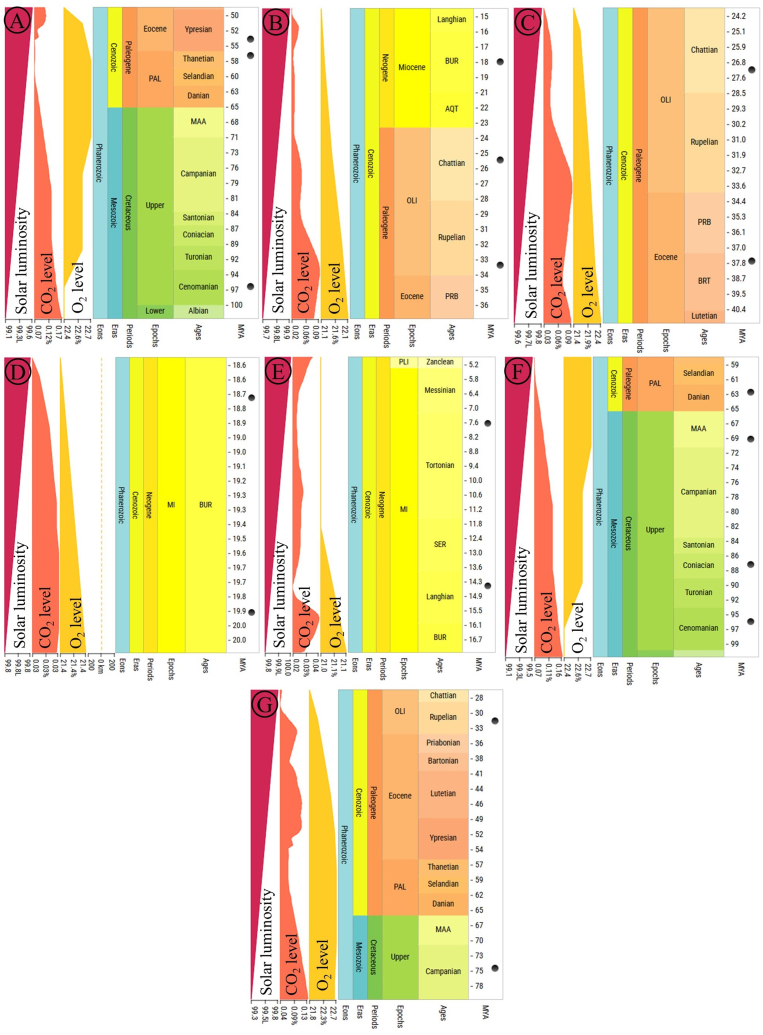
Pairwise divergence of different taxa to preset the calibration nodes for time tree calculation. A. *Eschscholzia californica* vs. *Chelidonium majus*, B. *Chelidonium majus* vs. *Dicranostigma* sp., C. *Meconopsis torquata* vs. *Papaver somniferum*, D. *Meconopsis racemosa* vs. *Meconopsis punicea*, E. *Papaver rhoeas* vs. *Papaver orientale*, F. *Fumaria officinalis* vs. *Hypecoum leptocarpum*, and G. *Dactylicapnos scandens* vs. *Lamprocapnos spectabilis.* Adjacent to the geological time scale, the time panels display solar luminosity, atomospheric CO_2_ and O_2_ levels. The solid black circles indicate molecular time estimates derived from the reference database of the TimeTree server.

**Fig. 10:**
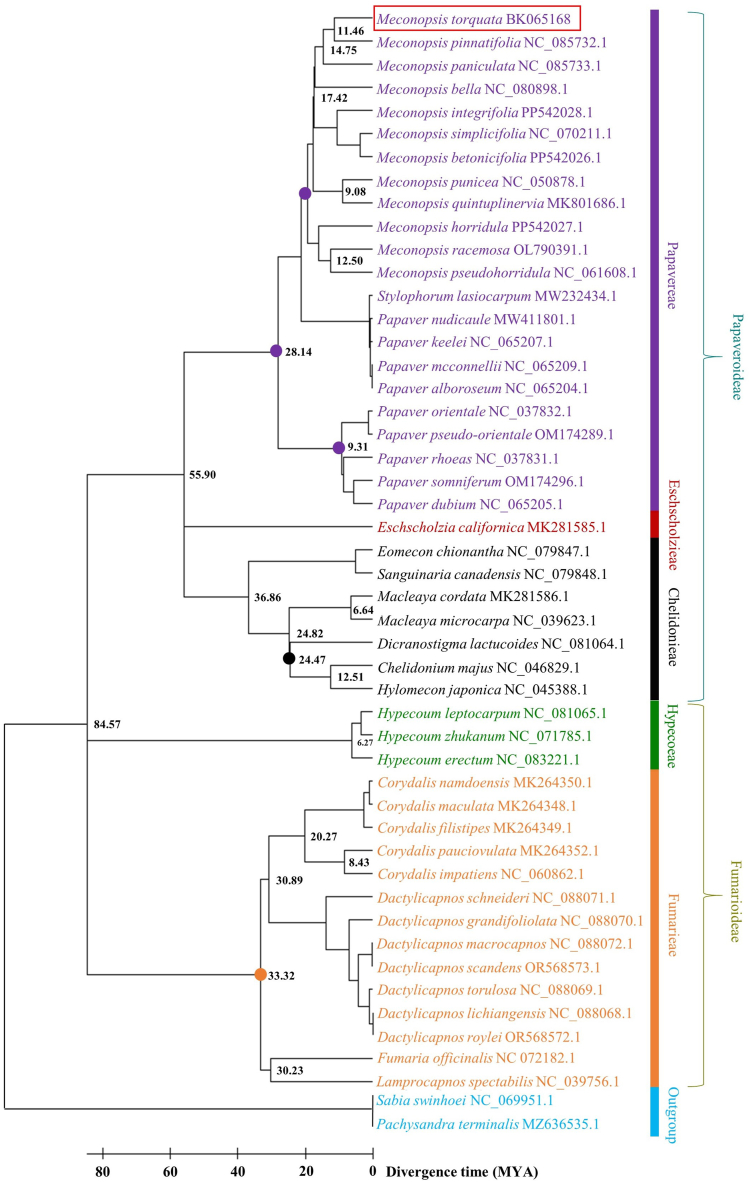
Plastome-wide molecular dating analysis of *M. torquata* within Papaveraceae. The multi-colored circles represent calibration nodes.

Calibration points were initially fixed for five nodes in the molecular dating analysis using the following seven pairs: (a) *Eschscholzia californica* vs. *Chelidonium majus*, (b) *Chelidonium majus* vs. *Dicranostigma* sp., (c) *Meconopsis torquata* vs. *Papaver somniferum*, (d) *Meconopsis racemosa* vs. *Meconopsis punicea*, (e) *Papaver rhoeas* vs. *Papaver orientale*, (f) *Fumaria officinalis* vs. *Hypecoum leptocarpum*, and (g) *Dactylicapnos scandens* vs. *Lamprocapnos spectabilis.* The median time estimated for these seven pairs was 57, 25.4, 32, 19.3, 11, 78, and 53 million years ago (MYA), respectively (Fig. 9). Using these calibrated nodes, molecular dating analysis revealed the origins of various plant tribes within Papaveraceae. Molecular dating analysis revealed that Papaveroideae originated around 55.90 MYA during the Ypresian age of the Paleogene period within the Cenozoic era. Fumarioideae was estimated to have originated around 84.57 MYA during the Coniacian age of the Cretaceous period within the Mesozoic era. Among the tribes of the subfamily Papaveroideae, Eschscholzieae was the most ancient (55.90 MYA) followed by Chelidonieae (36.86 MYA) and Papavereae (28.14 MYA). *M. torquata* was found to have diverged approximately 11.46 MYA during the Tortonian age of the Miocene epoch within the Cenozoic era. Time tree analysis of the subfamily Fumarioideae showed that the tribe Hypecoeae (84.57 MYA) is more ancient than the tribe Fumarieae (33.32 MYA). The tribe Fumarieae was estimated to have diverged during the Rupelian age of the Oligocene epoch within the Paleogene period of the Cenozoic era (Fig. 10).

## 4 Discussion

Rapid advancements in genomics and bioinformatics have significantly streamlined the sequencing and assembly of complete chloroplast genomes. Consequently, the number of complete cp genomes available in the public domain is exponentially increasing. This surge in available data offers an extensive and expanding repository of genetic information, facilitating a deeper understanding of plant evolution, phylogenetics, and functional genomics. Moreover, this study paves the way for exploring the genetic underpinnings of traits, evolutionary relationships, and adaptation mechanisms across a wide range of plant species. With continuous advances in technology, the accessibility and abundance of complete cp genome sequences are poised to drive breakthroughs in plant biology and related fields [31,32].

The cp genome size of *M. torquata* was consistent with that of several other members of the Papaveraceae family (Table 3). The genomic structure, gene organization, and features of genome divergence observed in *M. torquata* were in line with the typical characteristics of angiosperm plastomes [33,34]. The present investigation identified a total of 132 genes in *M. torquata*, comprising 87 PCGs, 37 tRNAs and eight rRNAs. A recent study on *M. bella* cp genome revealed 133 genes, comprising 88 PCGs, 37 tRNAs, and eight rRNAs [13]. Another study comparing cp genomes of 12 *Meconopsis* species revealed that PCGs ranged from 127 to 134, tRNAs from 29 to 37, and rRNAs from six to eight [35]. Our findings demonstrate similar gene numbers to other published *Meconopsis* cp genomes. The GC content showed a very similar scenario, showing higher levels in the IR region compared to the SSC and LSC regions (Table 1). This could be attributed to the selection pressures favoring GC-rich codons in genes encoding plastid proteins [36].

Genetic divergence was greater in the LSC and SSC regions than in the IR region in the *M. torquata* plastome, consistent with findings of previous study [35]. The IR regions are typically more conserved due to their role in maintaining structural stability and preventing recombination events that could lead to mutations. The increased divergence observed in the LSC and SSC regions of *M. torquata* plastome may be attributed to several factors, including higher mutation rates and reduced selective constraints compared to the IR regions. These regions often contain more genes involved in photosynthesis and other metabolic processes, which may evolve more rapidly due to environmental pressures and adaptation needs [37].

In the current study, we identified 120 SSRs in the *M. torquata* cp genome*.* SSRs are characterized by short, tandemly repeated DNA sequences that are typically composed of 1–6 nucleotide motifs. Their abundance in the *M. torquata* cp genome offers several advantages, including high polymorphism rates, codominant inheritance, and Mendelian segregation, making them potential markers for population genetics, phylogenetic studies, and molecular breeding. Furthermore, SSRs in the cp genome exhibit lower mutation rates than nuclear SSRs, enhancing their stability and reliability in evolutionary analyses [34]. Repeat structures were found to be closely parallel across the cp genomes of *Meconopsis* and *Papaver* (Fig. 3).

Codon usage analysis yielded results similar to those reported for the cp genome of *Chlorophytum* species, where tryptophan and methionine did not exhibit codon bias [38]. In our study, the cp genome of *M. torquata* also showed no codon bias for tryptophan or methionine, concordant with previous findings [33,39]. Comparative assessment of several other species, including *M. racemosa*, *M. punicea*, *Papaver somniferum*, *P. orientale*, and *P. rhoeas* demonstrated close similarity in total codon frequency, individual RSCU values, and cumulative RSCU values for different amino acids. Arginine, serine and leucine exhibited higher RSCU values, suggesting that these amino acids play a critical role in the survival and adaptation of these species, necessitating an optimized translation system for efficiency and accuracy. The observed codon usage bias in *Meconopsis* and *Papaver* genera may reflect adaptive response to ensure high expression levels and proper functioning of essential genes, aligning with the evolutionary strategies of these plants to thrive in their respective environments [40].

Junction site analysis revealed minimal size variation in the LSC, SSC and IR regions across the studied cp genomes, which was further supported by Nguyen et al. [34]. Sliding window analysis highlighted nucleotide diversity within *M. torquata* and its aligned chloroplast genomes, identifying hypervariable regions primarily located in the LSC and SSC regions. Comparable hypervariable regions have been documented in the LSC and SSC regions in the Asparagales order and Cinchonoideae subfamily [38,41]. In accordance with our findings, the SSC region contained the *ycf1* gene, while the LSC region harbored the *rps16* gene. The phylogenetic support for the barcode candidates observed in our study aligns with previously published reports [13,35,42]. Phylogenetic analysis of the NJ tree revealed that the *ycf1* gene was the most potential DNA barcode among the hypervariable genes (Fig. 7). This finding was congruent with the nucleotide diversity study, where the *ycf1* gene displayed the highest π value (0.1857) (Fig. 6). In several studies, the *ycf1* gene has been identified as one of the most promising barcodes for differentiating angiosperms [[43], [44], [45]]. Similar approaches have been followed in other studies to identify the most promising DNA barcodes [46,47].

The reconstructed phylogeny firmly positioned *M. torquata* with a very strong bootstrap support, confirming the accuracy of its cp genome assembly (Fig. 8). A recent study demonstrated tribal classification of Papaveraceae based on nuclear and plastid barcodes, where the tribe Papavereae showed a close relationship with the tribe Chelidonieae as compared to the tribe Eschscholzieae. Both Papveroideae and Fumarioideae subfamilies, along with all their respective tribes, were found to be monophyletic. In addition, the study identified *Papaver* as a polyphyletic genus and *Meconopsis* as a monophyletic genus [48], which align with our current investigation (Fig. 8). The polyphyletic nature of the genus *Papaver*, observed in both our current study and previous research, may underscore the urgent need for a comprehensive taxonomic revision of this genus. Furthermore, the recent shifting of *Papaver torquatum* (Prain) Christenh. & Byng to *Meconopsis torquata* Prain further highlights the blurred taxonomic boundaries between these genera. A meticulous taxonomic revision is essential to ensure both the genera as true monophyletic groups, thereby providing clearer and more accurate classifications [49].

Unlike a standardized DNA sequence fragment, the entire chloroplast genome harbors a greater number of mutation sites, rendering it a more efficient tool for identification purposes. By serving as a “superbarcode", the complete chloroplast genome has demonstrated successful applications in identifying taxa, particularly in the realm of medicinal species [33]. In an earlier study, the complete plastome proved effective as a superbarcode for differentiating several members of *Ligularia* [50]. Furthermore, the cp genome was used to differentiate *Chrysanthemum indicum*, highlighting the significance of the full plastome as a superbarcode for species identification [51]. Complete plastome sequences have also demonstrated superior discriminatory power in authenticating *Dendrobium* species, particularly in distinguishing *D. officinale* [52].

Plastome-wide molecular dating approach has been used to elucidate the divergence times of the members of the Myrtales order and *Urochloa* genus [53,54]. However, to date, no molecular dating studies targeting the full cp genomes of the member taxa of Papaveraceae have been reported. In the present study, molecular dating analysis revealed the emergence of the two subfamilies within Papaveraceae, with Fumarioideae (84.57 MYA) found to be more ancient than Papaveroideae (55.90 MYA) (Fig. 10). Fumarioideae was estimated to have originated during the Coniacian age of the Mesozoic era, while Papaveroideae originated during the Ypresian age of the Cenozoic era. During the Coniacian age of the Mesozoic era, angiosperms underwent significant diversification, rapidly spreading and evolving to occupy various ecological niches. They began to dominate many landscapes, often outcompeting gymnosperms and other plant groups. In the Ypresian age of the Cenozoic era, angiosperms continued their diversification, leading to the emergence of many advanced plant families. The climate was warm, facilitating the expansion of tropical and subtropical forests, which provided new environments for angiosperms to thrive and adapt [55,56].

## 5 Conclusion

The complete chloroplast genome of *Meconopsis torquata* was assembled *de novo* and annotated from Illumina paired-end reads. The cp genome exhibited a typical quadripartite structure, with size and organization similar to other angiosperm cp genomes. Identified repeat structures may serve as potential molecular markers for future identification and phylogenetic studies. Comparative phylogenomic analysis provided insights into genome organization and divergence across closely related species. Nucleotide diversity analysis identified hypervariable regions across the plastomes. Molecular phylogeny confirmed the proper clustering of the *M. troquata* plastome, with its member clades within Papaveraceae, supported by strong bootstrap values. Molecular dating analysis pinpointed the origin of *M. torquata* to the Tortonian age of the Miocene epoch within the Cenozoic era. The study will serve as a fundamental dataset for advancing plastome-wide phylogenetic and molecular dating research, providing significant insights into evolutionary trend and genetic diversity of *M. torquata* and related species within the family Papaveraceae.

## Data availability statement

All data and supporting information are provided in the article.

## Funding

This study did not receive support from any external funding sources.

## CRediT authorship contribution statement

**Sheikh Sunzid Ahmed:** Writing – original draft, Visualization, Software, Methodology, Investigation, Formal analysis, Data curation. **M. Oliur Rahman:** Writing – review & editing, Visualization, Validation, Supervision, Resources, Project administration, Methodology, Investigation, Data curation, Conceptualization.

## Declaration of competing interest

The authors hereby declare that there is no any conflicting interest concerning financial or personal issues that might have influenced the work presented in this paper.
