# Supplementary material for: Deciphering the complete chloroplast genome sequence of Meconopsis torquata Prain: Insights into genome structure, comparative analysis and phylogenetic relationship
Source: Heliyon. 2024 Aug 13;10(16):e36204. doi: 10.1016/j.heliyon.2024.e36204 (PMC11367419; doi:10.1016/j.heliyon.2024.e36204)
Supplement: Multimedia component 1 [file mmc1.docx]

Manuscript. Number.: **HELIYON-D-24-24389**

**Deciphering the complete chloroplast genome sequence of *Meconopsis torquata* Prain: insights into genome structure, comparative analysis and phylogenetic relationship**

SHEIKH SUNZID AHMED AND M. OLIUR RAHMAN*

*Department of Botany, Faculty of Biological Sciences, University of Dhaka, Dhaka 1000, Bangladesh*

**Supporting information file**

**Table S1**

Per base sequence quality reports of the forward reads generated via FASTQC tool.

| Base | Mean | Median | Lower Quartile | Upper Quartile | 10th Percentile | 90th Percentile |
| --- | --- | --- | --- | --- | --- | --- |
| 1 | 35.23808 | 37 | 37 | 37 | 37 | 37 |
| 2 | 35.83639 | 37 | 37 | 37 | 37 | 37 |
| 3 | 36.02644 | 37 | 37 | 37 | 37 | 37 |
| 4 | 36.11373 | 37 | 37 | 37 | 37 | 37 |
| 5 | 36.13536 | 37 | 37 | 37 | 37 | 37 |
| 6 | 36.15303 | 37 | 37 | 37 | 37 | 37 |
| 7 | 36.12151 | 37 | 37 | 37 | 37 | 37 |
| 8 | 36.15632 | 37 | 37 | 37 | 37 | 37 |
| 9 | 36.18451 | 37 | 37 | 37 | 37 | 37 |
| 10-14 | 36.26959 | 37 | 37 | 37 | 37 | 37 |
| 15-19 | 36.47393 | 37 | 37 | 37 | 37 | 37 |
| 20-24 | 36.52501 | 37 | 37 | 37 | 37 | 37 |
| 25-29 | 36.33079 | 37 | 37 | 37 | 37 | 37 |
| 30-34 | 36.30914 | 37 | 37 | 37 | 37 | 37 |
| 35-39 | 36.34727 | 37 | 37 | 37 | 37 | 37 |
| 40-44 | 36.35472 | 37 | 37 | 37 | 37 | 37 |
| 45-49 | 36.36852 | 37 | 37 | 37 | 37 | 37 |
| 50-54 | 36.36736 | 37 | 37 | 37 | 37 | 37 |
| 55-59 | 36.35976 | 37 | 37 | 37 | 37 | 37 |
| 60-64 | 36.35492 | 37 | 37 | 37 | 37 | 37 |
| 65-69 | 36.32928 | 37 | 37 | 37 | 37 | 37 |
| 70-74 | 36.30543 | 37 | 37 | 37 | 37 | 37 |
| 75-79 | 36.28473 | 37 | 37 | 37 | 37 | 37 |
| 80-84 | 36.25751 | 37 | 37 | 37 | 37 | 37 |
| 85-89 | 36.22683 | 37 | 37 | 37 | 37 | 37 |
| 90-94 | 36.19396 | 37 | 37 | 37 | 37 | 37 |
| 95-99 | 36.15727 | 37 | 37 | 37 | 37 | 37 |
| 100-104 | 36.12402 | 37 | 37 | 37 | 37 | 37 |
| 105-109 | 36.09101 | 37 | 37 | 37 | 37 | 37 |
| 110-114 | 36.05578 | 37 | 37 | 37 | 37 | 37 |
| 115-119 | 36.00678 | 37 | 37 | 37 | 37 | 37 |
| 120-124 | 35.97172 | 37 | 37 | 37 | 37 | 37 |
| 125-129 | 35.9278 | 37 | 37 | 37 | 37 | 37 |
| 130-134 | 35.88471 | 37 | 37 | 37 | 37 | 37 |
| 135-139 | 35.83624 | 37 | 37 | 37 | 37 | 37 |
| 140-144 | 35.78378 | 37 | 37 | 37 | 37 | 37 |
| 145-149 | 35.73262 | 37 | 37 | 37 | 37 | 37 |
| 150 | 35.69499 | 37 | 37 | 37 | 37 | 37 |

**Table S2**

Per base sequence quality reports of the reverse reads generated via FASTQC tool.

| Base | Mean | Median | Lower Quartile | Upper Quartile | 10th Percentile | 90th Percentile |
| --- | --- | --- | --- | --- | --- | --- |
| 1 | 33.76508039 | 37 | 37 | 37 | 20 | 37 |
| 2 | 35.30284952 | 37 | 37 | 37 | 37 | 37 |
| 3 | 35.57868527 | 37 | 37 | 37 | 37 | 37 |
| 4 | 35.75016991 | 37 | 37 | 37 | 37 | 37 |
| 5 | 35.82204086 | 37 | 37 | 37 | 37 | 37 |
| 6 | 35.89556175 | 37 | 37 | 37 | 37 | 37 |
| 7 | 35.89185323 | 37 | 37 | 37 | 37 | 37 |
| 8 | 35.92451948 | 37 | 37 | 37 | 37 | 37 |
| 9 | 35.97274635 | 37 | 37 | 37 | 37 | 37 |
| 10-14 | 36.08162768 | 37 | 37 | 37 | 37 | 37 |
| 15-19 | 36.15566962 | 37 | 37 | 37 | 37 | 37 |
| 20-24 | 36.19042478 | 37 | 37 | 37 | 37 | 37 |
| 25-29 | 35.87070508 | 37 | 37 | 37 | 37 | 37 |
| 30-34 | 35.84049583 | 37 | 37 | 37 | 37 | 37 |
| 35-39 | 35.88302754 | 37 | 37 | 37 | 37 | 37 |
| 40-44 | 35.90757101 | 37 | 37 | 37 | 37 | 37 |
| 45-49 | 35.93060398 | 37 | 37 | 37 | 37 | 37 |
| 50-54 | 35.94327248 | 37 | 37 | 37 | 37 | 37 |
| 55-59 | 35.95306921 | 37 | 37 | 37 | 37 | 37 |
| 60-64 | 35.94992524 | 37 | 37 | 37 | 37 | 37 |
| 65-69 | 35.94423786 | 37 | 37 | 37 | 37 | 37 |
| 70-74 | 35.91972003 | 37 | 37 | 37 | 37 | 37 |
| 75-79 | 35.88415389 | 37 | 37 | 37 | 37 | 37 |
| 80-84 | 35.87349024 | 37 | 37 | 37 | 37 | 37 |
| 85-89 | 35.83817222 | 37 | 37 | 37 | 37 | 37 |
| 90-94 | 35.80923691 | 37 | 37 | 37 | 37 | 37 |
| 95-99 | 35.76040139 | 37 | 37 | 37 | 37 | 37 |
| 100-104 | 35.71289063 | 37 | 37 | 37 | 37 | 37 |
| 105-109 | 35.66774059 | 37 | 37 | 37 | 37 | 37 |
| 110-114 | 35.61568229 | 37 | 37 | 37 | 37 | 37 |
| 115-119 | 35.55250832 | 37 | 37 | 37 | 37 | 37 |
| 120-124 | 35.49875328 | 37 | 37 | 37 | 37 | 37 |
| 125-129 | 35.42790776 | 37 | 37 | 37 | 37 | 37 |
| 130-134 | 35.36534162 | 37 | 37 | 37 | 37 | 37 |
| 135-139 | 35.29385367 | 37 | 37 | 37 | 37 | 37 |
| 140-144 | 35.19966267 | 37 | 37 | 37 | 37 | 37 |
| 145-149 | 35.12231474 | 37 | 37 | 37 | 37 | 37 |
| 150 | 35.06856138 | 37 | 37 | 37 | 37 | 37 |

**Table S3**

Longer repeats position in the cp genome of *M. torquata* identified using REPuter server.

| Sl. No. | Repeat size | Repeat type | Repeat position 1 | Repeat position 2 | E-value |
| --- | --- | --- | --- | --- | --- |
| 1 | 52 | P | 74129 | 74129 | 3.26E-22 |
| 2 | 31 | P | 63783 | 63783 | 1.43E-09 |
| 3 | 29 | P | 8297 | 8297 | 2.29E-08 |
| 4 | 28 | P | 4483 | 4483 | 9.17E-08 |
| 5 | 24 | P | 285 | 285 | 2.35E-05 |
| 6 | 24 | P | 35603 | 35603 | 2.35E-05 |
| 7 | 23 | R | 109787 | 109787 | 9.39E-05 |
| 8 | 23 | P | 114921 | 114921 | 9.39E-05 |
| 9 | 21 | F | 8302 | 8302 | 1.50E-03 |
| 10 | 21 | P | 35537 | 35537 | 1.50E-03 |
| 11 | 21 | F | 47941 | 47941 | 1.50E-03 |
| 12 | 21 | R | 80292 | 80292 | 1.50E-03 |
| 13 | 21 | R | 81515 | 81515 | 1.50E-03 |
| 14 | 21 | P | 116643 | 116643 | 1.50E-03 |
| 15 | 20 | P | 6492 | 6492 | 6.01E-03 |
| 16 | 20 | P | 47658 | 47658 | 6.01E-03 |
| 17 | 20 | P | 52244 | 52244 | 6.01E-03 |
| 18 | 20 | F | 52244 | 52244 | 6.01E-03 |
| 19 | 20 | P | 66030 | 66030 | 6.01E-03 |
| 20 | 19 | P | 5765 | 5765 | 2.40E-02 |
| 21 | 19 | R | 7273 | 7273 | 2.40E-02 |
| 22 | 19 | F | 9998 | 9998 | 2.40E-02 |
| 23 | 19 | F | 47658 | 47658 | 2.40E-02 |
| 24 | 19 | P | 47658 | 47658 | 2.40E-02 |
| 25 | 19 | F | 102490 | 102490 | 2.40E-02 |
| 26 | 19 | P | 102490 | 102490 | 2.40E-02 |
| 27 | 19 | P | 103346 | 103346 | 2.40E-02 |
| 28 | 19 | F | 133843 | 133843 | 2.40E-02 |
| 29 | 18 | C | 252 | 252 | 9.62E-02 |
| 30 | 18 | P | 4557 | 4557 | 9.62E-02 |
| 31 | 18 | P | 5767 | 5767 | 9.62E-02 |
| 32 | 18 | P | 29072 | 29072 | 9.62E-02 |
| 33 | 18 | P | 57515 | 57515 | 9.62E-02 |
| 34 | 18 | C | 65319 | 65319 | 9.62E-02 |
| 35 | 18 | R | 65319 | 65319 | 9.62E-02 |
| 36 | 18 | C | 80292 | 80292 | 9.62E-02 |
| 37 | 18 | P | 80295 | 80295 | 9.62E-02 |
| 38 | 18 | F | 91135 | 91135 | 9.62E-02 |
| 39 | 18 | P | 91135 | 91135 | 9.62E-02 |
| 40 | 18 | P | 91159 | 91159 | 9.62E-02 |
| 41 | 18 | P | 102087 | 102087 | 9.62E-02 |
| 42 | 18 | F | 102087 | 102087 | 9.62E-02 |
| 43 | 18 | F | 108297 | 108297 | 9.62E-02 |
| 44 | 18 | P | 113285 | 113285 | 9.62E-02 |
| 45 | 18 | F | 125286 | 125286 | 9.62E-02 |
| 46 | 18 | P | 128893 | 128893 | 9.62E-02 |
| 47 | 18 | F | 146031 | 146031 | 9.62E-02 |
| 48 | 17 | C | 255 | 255 | 3.85E-01 |
| 49 | 17 | R | 255 | 255 | 3.85E-01 |

**Table S4**

Codon-anticodon recognition pattern and codon usage for the *Meconopsis torquata* chloroplast genome.

| Codon | Amino acid | Count | RSCU | trnA | Codon | Amino acid | Count | RSCU | trnA |
| --- | --- | --- | --- | --- | --- | --- | --- | --- | --- |
| UUU | F | 2051 | 1.19 | *trnF-GAA* | UAU | Y | 1340 | 1.32 | *trnY-GUA* |
| UUC | F | 1397 | 0.81 |  | UAC | Y | 687 | 0.68 |  |
| UUA | L | 1004 | 1.20 | *trnL-UAA* | UAA | * | 1062 | 1.10 |  |
| UUG | L | 1028 | 1.23 | *trnL-CAA* | UAG | * | 781 | 0.81 |  |
| CUU | L | 1051 | 1.26 | *trnL-UAG* | CAU | H | 965 | 1.43 | *trnH-GUG* |
| CUC | L | 687 | 0.82 |  | CAC | H | 388 | 0.57 |  |
| CUA | L | 768 | 0.92 |  | CAA | Q | 1047 | 1.40 | *trnQ-UUG* |
| CUG | L | 478 | 0.57 |  | CAG | Q | 446 | 0.60 |  |
| AUU | I | 1641 | 1.21 | *trnI-GAU* | AAU | N | 1592 | 1.35 | *trnN-GUU* |
| AUC | I | 1081 | 0.80 |  | AAC | N | 762 | 0.65 |  |
| AUA | I | 1344 | 0.99 | *trnI-CAU* | AAA | K | 1987 | 1.33 | *trnK-UUU* |
| AUG | M | 862 | 1.00 | *trnfM-CAU* | AAG | K | 1010 | 0.67 |  |
| GUU | V | 800 | 1.40 | *trnV-GAC* | GAU | D | 1014 | 1.39 | *trnD-GUC* |
| GUC | V | 429 | 0.75 |  | GAC | D | 441 | 0.61 |  |
| GUA | V | 662 | 1.16 | *trnV-UAC* | GAA | E | 1278 | 1.32 | *trnE-UUC* |
| GUG | V | 398 | 0.70 |  | GAG | E | 652 | 0.68 |  |
| UCU | S | 1161 | 1.45 | *trnS-GGA* | UGU | C | 673 | 1.17 | *trnC-GCA* |
| UCC | S | 971 | 1.21 |  | UGC | C | 475 | 0.83 |  |
| UCA | S | 931 | 1.16 | *trnS-UGA* | UGA | * | 1042 | 1.08 |  |
| UCG | S | 573 | 0.71 |  | UGG | W | 702 | 1.00 | *trnW-CCA* |
| CCU | P | 707 | 1.09 | *trnP-UGG* | CGU | R | 401 | 0.71 | *trnR-ACG* |
| CCC | P | 699 | 1.08 |  | CGC | R | 255 | 0.45 | *trnR-UCU* |
| CCA | P | 763 | 1.18 |  | CGA | R | 559 | 0.98 |  |
| CCG | P | 414 | 0.64 |  | CGG | R | 408 | 0.72 |  |
| ACU | T | 651 | 1.10 | *trnT-GGU* | AGA | R | 1095 | 1.93 |  |
| ACC | T | 636 | 1.08 |  | AGG | R | 692 | 1.22 |  |
| ACA | T | 687 | 1.16 | *trnT-UGU* | AGU | S | 689 | 0.86 | *trnS-GCU* |
| ACG | T | 385 | 0.65 |  | AGC | S | 494 | 0.62 |  |
| GCU | A | 514 | 1.30 | *trnA-UGC* | GGU | G | 573 | 0.99 | *trnG-GCC* |
| GCC | A | 341 | 0.87 |  | GGC | G | 379 | 0.65 |  |
| GCA | A | 454 | 1.15 |  | GGA | G | 796 | 1.37 | *trnG-UCC* |
| GCG | A | 267 | 0.68 |  | GGG | G | 576 | 0.99 |  |

__________________________________________________

Total Codon: 64; Total unique amino acids: 20; Total codon frequency:51,096


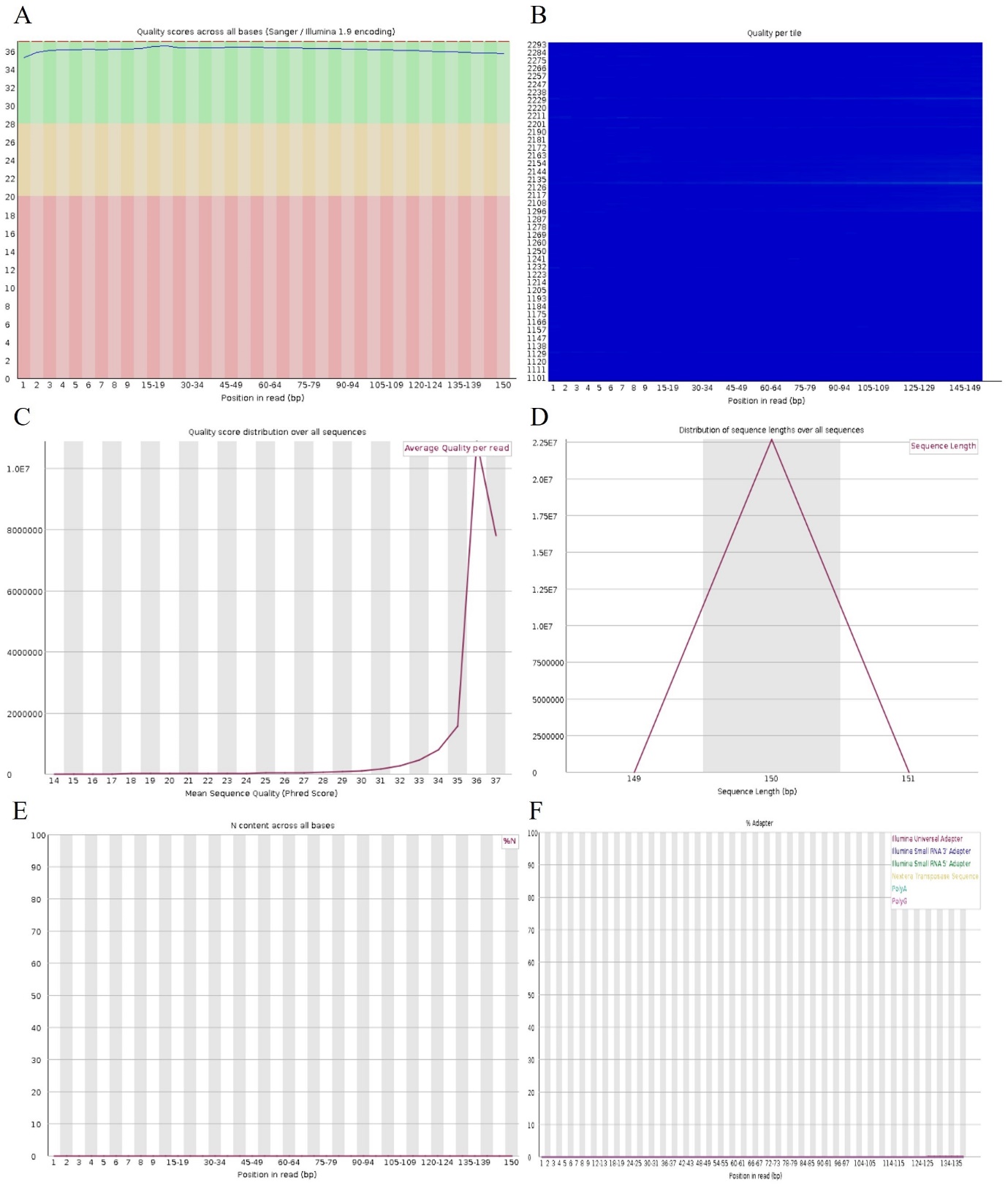


**Fig. S1.** Quality control scores of the forward reads generated by FASTQC tool. A. Per base sequence quality, B. Per tile sequence quality, C. Per sequence quality scores, D. Sequence length distribution, E. Per base N content and F. Adapter content.


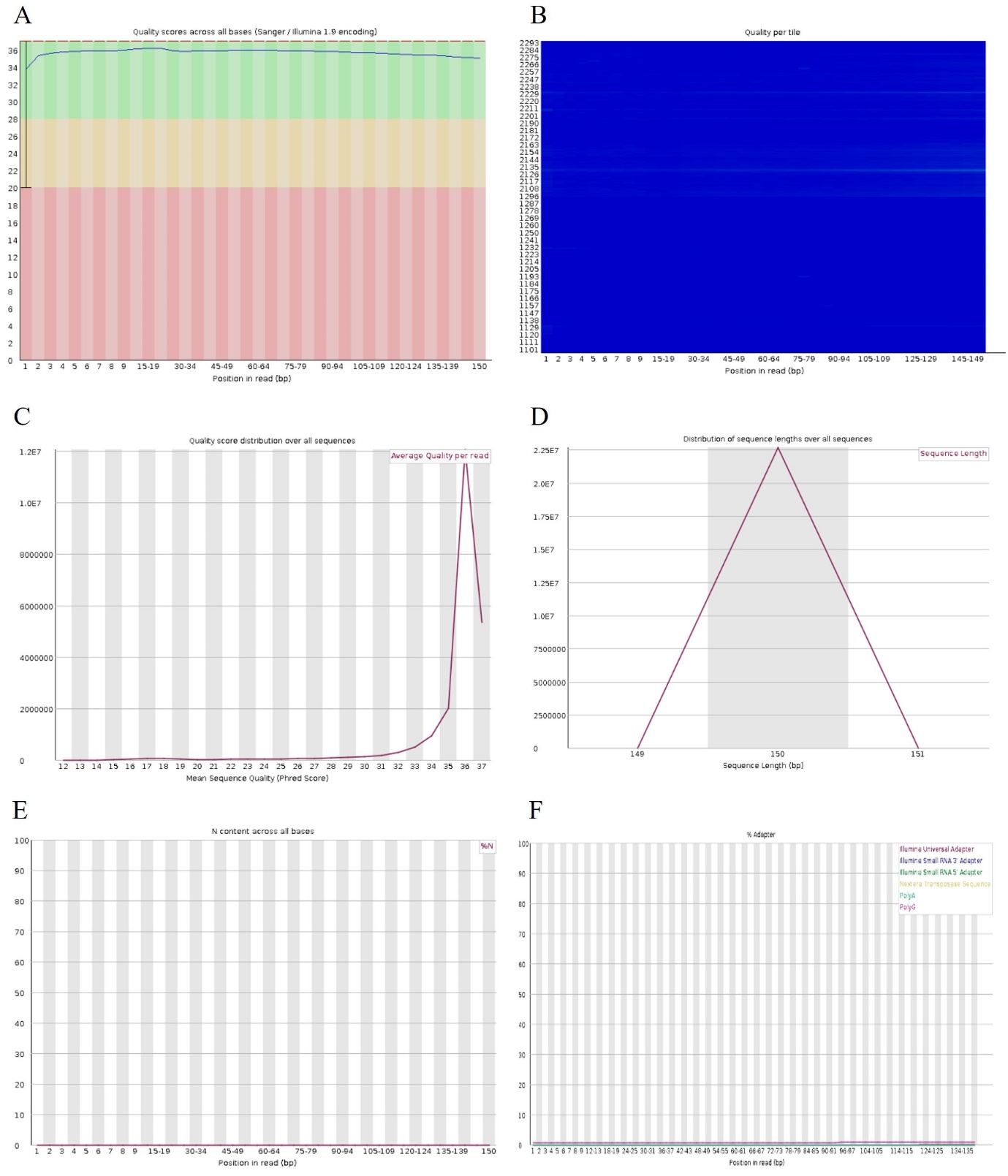


**Fig. S2.** Quality control scores of the reverse reads generated by FASTQC tool. A. Per base sequence quality, B. Per tile sequence quality, C. Per sequence quality scores, D. Sequence length distribution, E. Per base N content and F. Adapter content.
